# Identification of lncRNAs associated with early‐stage breast cancer and their prognostic implications

**DOI:** 10.1002/1878-0261.12489

**Published:** 2019-05-08

**Authors:** Arunagiri Kuha Deva Magendhra Rao, Krishna Patel, Suneetha Korivi Jyothiraj, Balaiah Meenakumari, Shirley Sundersingh, Velusami Sridevi, Thangarajan Rajkumar, Akhilesh Pandey, Aditi Chatterjee, Harsha Gowda, Samson Mani

**Affiliations:** ^1^ Department of Molecular Oncology Cancer Institute (WIA) Chennai India; ^2^ Institute of Bioinformatics Bangalore India; ^3^ Amrita School of Biotechnology Amrita Vishwa Vidyapeetham Kollam India; ^4^ Department of Oncopathology Cancer Institute (WIA) Chennai India; ^5^ Department of Surgical Oncology Cancer Institute (WIA) Chennai India; ^6^ Department of Laboratory Medicine and Pathology Mayo Clinic Rochester NY USA; ^7^ Center for Individualized Medicine Mayo Clinic Rochester NY USA; ^8^ Manipal Academy of Higher Education (MAHE) Manipal India; ^9^ Center for Molecular Medicine National Institute of Mental Health and Neurosciences (NIMHANS) Bangalore India; ^10^ QIMR Berghofer Medical Research Institute Brisbane Australia

**Keywords:** ADAMTS9‐AS2, breast cancer, FAM83H‐AS1, long noncoding RNAs, ncRNAs, RNA sequencing

## Abstract

Breast cancer is the most common malignancy among women, with the highest incidence rate worldwide. Dysregulation of long noncoding RNAs during the preliminary stages of breast carcinogenesis is poorly understood. In this study, we performed RNA sequencing to identify long noncoding RNA expression profiles associated with early‐stage breast cancer. RNA sequencing was performed on six invasive ductal carcinoma (IDC) tissues along with paired normal tissue samples, seven ductal carcinoma *in situ* tissues, and five apparently normal breast tissues. We identified 375 differentially expressed lncRNAs (DElncRNAs) in IDC tissues compared to paired normal tissues. Antisense transcripts (~ 58%) were the largest subtype among DElncRNAs. About 20% of the 375 DElncRNAs were supported by typical split readings leveraging their detection confidence. Validation was performed in *n* = 52 IDC and paired normal tissue by qRT‐PCR for the identified targets (ADAMTS9‐AS2, EPB41L4A‐AS1, WDFY3‐AS2, RP11‐295M3.4, RP11‐161M6.2, RP11‐490M8.1, CTB‐92J24.3, and FAM83H‐AS1). We evaluated the prognostic significance of DElncRNAs based on TCGA datasets and report that overexpression of FAM83H‐AS1 was associated with patient poor survival. We confirmed that the downregulation of ADAMTS9‐AS2 in breast cancer was due to promoter hypermethylation through *in vitro* silencing experiments and pyrosequencing.

**Abbreviations**
DCIS: ductal carcinoma *in situ*
IDC: invasive ductal carcinoma
lincRNA: long intergenic noncoding RNA
lncRNA: long noncoding RNAs
PCA: principal component analysis
PCC: Pearson′s correlation coefficient
TNBC: triple‐negative breast cancers

## 1 Introduction

Breast cancer is the most common cancer among women (ASR‐43.1) with highest mortality rates (Ferlay *et al*., 2013). Breast cancer is broadly classified into noninvasive ductal carcinoma *in situ* (DCIS) and invasive ductal carcinoma (IDC). Understanding the mechanism of breast carcinogenesis at genetic and transcriptional level can aid in characterization of DCIS or early‐stage IDC tumors. Gene expression signatures are used to classify IDC subtypes of hormone receptor‐positive (estrogen and progesterone receptors), that is, luminal A and B, and hormone receptor‐negative HER2 and basal‐like (Perou *et al*., 2000; Sorlie *et al*., 2001) breast cancer subtypes. Next‐generation sequencing has enabled global profiling of mRNAs and noncoding RNAs (ncRNAs) including long ncRNAs (lncRNAs) and microRNAs. lncRNAs have gained immense importance in gene regulation and are known to play an important role in cancer development and prognosis (Huarte, 2015; Prensner and Chinnaiyan, 2011; Rao *et al*., 2017). Understanding the divergent expression of lncRNAs in early‐stage breast tumors can help elucidate its functional role in carcinogenesis.

Specific lncRNAs signatures are known to be associated with different molecular subtypes of breast cancer. DSCAM‐AS1 was identified specifically in ER‐positive breast tumors and shown to increase aggression and drug resistance (Niknafs *et al*., 2016). Similarly, AFAP1‐AS1 was predominantly found to be dysregulated in HER2 and triple‐negative breast cancers (TNBC) (Shen *et al*., 2015; Yang *et al*., 2016a). H19 was identified to be overexpressed in ER/PR‐positive breast adenomas, and BC200 was implicated to be distinctly elevated in benign tumors and not in invasive subtypes and hence is of prognostic significance (Adriaenssens *et al*., 1998; Iacoangeli *et al*., 2004). HOTAIR was demonstrated to gain activity in BRCA1‐mutated tumors. In a normal cell, BRCA1 competes with HOTAIR in binding to EZH2 of PRC2 (Wang *et al*., 2013). The functional characteristics of certain lncRNAs, such as UCA1, GAS5, and XIST, have established them as breast cancer‐associated tumor suppressors, while HOTAIR, TINCR, and DSCAM‐AS1 are known as oncogenic lncRNAs (Wang *et al*., 2017; Xu *et al*., 2017). Support vector machine‐based prediction of breast cancer intrinsic subtype using lncRNA expression profile and PAM50 gene signature using TCGA datasets was recently proposed as an improved prediction model (Zhang *et al*., 2018).

Despite known association of lncRNA expression with molecular subtype, recently reported lncRNAs have emerging role in relevant signaling or druggable pathways. lncRNA CYTOR was reported to be associated with breast cancer progression through EGFR signaling pathway (Van Grembergen *et al*., 2016). NKILA was observed to promote heterotrimeric complex formation (p50/p60/IκB) and inhibit IκB phosphorylation, thus regulating NF‐κB signaling (Liu *et al*., 2015). LINK‐A was reported to aid in stabilizing HIF1α in normoxic conditions of TNBC. Through BRK/PTK6 activation and phosphorylation of HIF1α, LINK‐A substantiates its kinase activation and cancer signaling potential (Lin *et al*., 2016). Alternatively, breast cancer‐associated lncRNAs important in drug targeting pathways can also be useful prognostic biomarkers. In the present study, we have done RNA sequencing in early‐stage tumors (stage I–IIA IDC, DCIS) and noncancerous breast tissue samples to identify lncRNAs that play a role in early‐stage breast cancer. We speculate that aberrant expression of lncRNAs could be an early event in breast cancer development, and hence, the study was aimed to identify dysregulated lncRNAs and the mechanism of dysregulation in breast cancer.

## 2 Materials and methods

### 2.1 Study population and sample classification

The study cohort includes patients diagnosed and treated for breast cancer at Cancer Institute (WIA), Chennai, Tamil Nadu, India. These patients were histologically confirmed of invasive ductal carcinoma (IDC—stage I–II A) and DCIS. Apparently, normal breast tissues were obtained from patients undergoing surgery for breast conditions other than malignancy. Samples having > 70% for cancer cells following histopathological examination were included in the study. Paired normal and apparently normal tissues completely free of tumor cells were selected and kept frozen (−80 °C) until further processing. Total RNA sequencing was done for 24 samples, that is, tumor (*n* = 6), paired normal (matched normal; *n* = 6), DCIS (*n* = 7), and apparently normal (*n* = 5). Validation cohort of IDC (*n* = 52) and corresponding paired normal tissue were used to gauge candidate lncRNAs. The clinicopathological features of patients in the discovery and validation cohort are detailed in Table S1. All patients were informed about the study, and their written consent for participation was obtained. The Institutional Ethical Committee approved the study and the protocol duly conforming the guidelines set by the Declaration of Helsinki.

### 2.2 RNA isolation and library preparation

Total RNA was isolated from frozen tissues using TRIzol method and purification by NucleoSpin RNA Isolation Kit (Macherey‐Nagel, Düren, Germany), which includes an on‐column DNase treatment. The quality and quantity of total RNA was evaluated through 2100 Bioanalyzer (Agilent Technologies, Santa Clara, CA, USA). Ribosomal RNA was depleted (EpiGentek, USA), and cDNA library was prepared using Illumina TruSeq Stranded Total RNA Library Prep Kit. The library profile was verified using 2100 Bioanalyzer (Agilent Technologies). Subsequent RNA sequencing of cDNA libraries with paired‐end reads (2 × 100 bps reads) was performed according to the standard Illumina protocol using HiSeq 2500 sequencing platform.

### 2.3 RNA sequencing and data analysis

Raw reads were assessed for Phred quality using fastqc (Andrews, 2010), and low bases and adaptor sequences were trimmed off using Fqtrim (Pertea, 2015) retaining reads of length ≥ 75 bases. Clean reads were aligned against human reference genome (GRCh38 assembly) with Gencode V24 annotation using hisat2 (Baruzzo *et al*., 2017) with default parameters. Exon centric read counts were obtained from binary alignment map using HTSeq (Anders *et al*., 2015) using the script ‘htseq count’ for all samples independently. lncRNAs identified with ≥ 15 reads in at least three samples per cohort, that is, IDC, paired normal, DCIS, and apparent normal were further investigated for differential expression using DESeq (Anders and Huber, 2010). Read counts obtained from HTSeq were normalized using ‘estimateSizeFactors’ variance and were modeled using ‘estimateDispersions’. The differentially expressed genes were computed using ‘nbinomTest’ functions of DESeq. Significant differential expression was defined if |log_2_ (fold‐change)| > 1 and q‐value (Bonferroni and Benjamini–Hochberg adjusted *P* value) < 0.1. Expression profile of lncRNAs from TCGA breast cancer dataset (TCGA‐BRCA; *n* = 837 invasive tumors and *n* = 105 normal samples) was used for survival analysis (Li *et al*., 2015). Kaplan–Meier plots for differentially expressed lncRNAs (DElncRNAs) were generated for tumor stages as well as molecular subtypes and evaluated using log‐rank test.

### 2.4 lncRNA‐mRNA co‐expression network analysis

Pearson′s correlation coefficient (PCC) was used to determine linear correlation between mRNA and lncRNA expression profiles using r package. DElncRNAs DElncRNAs ‐mRNA pairs with |PCC| ≥ 0.9 were considered for network analysis using string v10 (Szklarczyk *et al*., 2015) with organism ‘Human’ as backend database and Cytoscape (Shannon *et al*., 2003).

### 2.5 Real‐time quantitative PCR

Total RNA of 500 ng was used for preparing cDNA libraries using QuantiTect Reverse Transcription Kit (Qiagen, Hilden, Germany). Gene expression was estimated by QuantStudio 12K Flex Real‐Time PCR System using TaqMan™ gene expression assays (Applied Biosystems, Thermo Fisher Scientific, Waltham, Massachusetts, USA) containing primers and probes specific for lncRNA and GAPDH. The expression values were calculated using the $2^{\Delta C_{t}}$ method (Δ*C*_t_ = Δ*C*_t_ target gene‐Δ*C*_t_ reference gene).

### 2.6 siRNA‐mediated knock‐down of DNMT1

Expression of ADAMTS9‐AS2 was evaluated in MDAMB‐231 and MCF7 cells. The cells were cultured in Dulbecco's modified Eagle's medium with 10% FBS at 37 °C. Knock‐down was carried out using Lipofectamine 3000 (Life Technologies, Carlsbad, CA, USA), siRNA targeting DNMT1 (Ambion, USA) with cells maintained in Opti‐MEM (Life Technologies) during and after transfection. Transfected cells were collected after 48 and 72 h for total RNA and DNA isolation.

### 2.7 DNA extraction, Bisulfite treatment, and pyrosequencing

Genomic DNA was extracted from tissues and cultured MDAMB‐231 and MCF7 cells using NucleoSpin Kit (Macherey‐Nagel, GmbH). About 500 ng of DNA was used for bisulfite treatment following manufacturer's protocol of EZ DNA Methylation‐Gold Kit (Zymo Research, Irvine, CA, USA). Bisulfite‐treated DNA was amplified using inventoried PyroMark CpG assay *Hs_AC132007.1_01_PM* (Qiagen, GmbH) with primers spanning ADAMTS9‐AS2 promoter region. The amplified fragment was sequenced using pyromark Q48 Autoprep (Qiagen, GmbH) and analyzed by pyromark q24 software v 2.0.7.

### 2.8 Statistical analyses

graphpad prism7 (GraphPad Prism Software Inc., San Diego, CA, USA) was used for evaluating qRT‐PCR gene expression data. Student's *t*‐test was used for pairwise analysis of tumor and paired normal samples. Welch correction was done if significant difference in variance was observed and Wilcoxon rank sum test was applied whenever non‐Gaussian distribution was followed.

## 3 Results

### 3.1 Expression profile of lncRNAs in ductal carcinoma *in situ* and invasive ductal carcinoma

**Figure 1:**
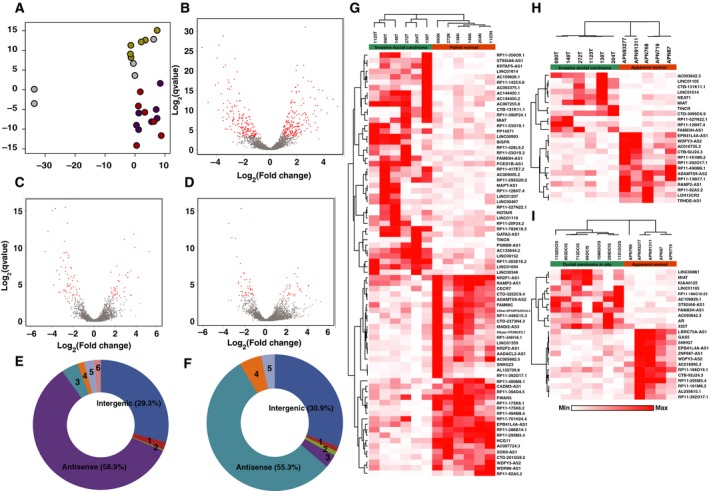
Summary of DElncRNAs identified in DCIS and early‐stage breast cancer. (A) PCA based on lncRNA expression profile to demonstrate distinct segregation of tissues of various pathological types. Color legend: Apparent normal: yellow; DCIS: purple; Paired normal: green; IDC: red samples. (B) Volcano plot represents the expression pattern of lncRNA in IDC vs. paired normal samples. (C) Volcano plot represents the expression pattern of lncRNA in IDC vs. apparent normal samples. (D) Volcano plot represents the expression pattern of lncRNA in DCIS vs. apparent normal samples. (E) Pie chart representing DElncRNA subtypes in IDC vs. paired normal samples [1—intron overlapping (1.9%); 2—noncoding transcript (0.3%); 3—TEC (0.4%); 4—sense overlapping (1.5%); 5—processed transcript (2.4%); 6—completely intronic (1.6%)]. (F)Pie chart representing DElncRNA subtypes in IDC vs. apparent normal samples [1—intron overlapping (1.1%); 2—completely intronic (1.1%); 3—TEC (3.2%); 4—processed transcript (5.3%); 5—sense overlapping (3.2%)]. (G) Heatmap with supervised clustering represents the expression trend of DElncRNAs in IDC vs. paired normal samples. (H) Heatmap with supervised clustering represents the expression trend of DElncRNAs in IDC vs. apparent normal samples. (I) Heatmap with supervised clustering represents the expression trend of DElncRNAs in DCIS vs. apparent normal samples.

RNA sequencing resulted in generation of ~ 89 million reads per sample with ~ 87.24% alignment against human genome build Hg38. We identified ~ 2 689 lncRNAs and ~ 18 132 mRNAs with ≥ 15 reads in at least three samples per cohort (Table 1, Table S2). In agreement with previous reports, lncRNAs were expressed at comparatively lower levels than mRNAs (Fig. S1A–D). Principal component analysis (PCA) plots based on lncRNA quantification showed distinct segregation of tumors (IDC and DCIS) from paired and apparent normal samples reflecting the characteristic variation of lncRNA expression profile (Fig. 1A, Fig. S1E). Differential expression analysis was performed between IDC, DCIS, and control samples in four categories, that is, IDC vs. paired normal (TN), IDC vs. apparent normal (TA), DCIS vs. apparent normal (DA), and IDC vs. DCIS (TD); (Fig 1B–D, Fig S2, Table S3‐S6).

**Table 1:** Number of DElncRNAs in DCIS and early‐stage breast cancer

Comparison set	lncRNA			
	Overexpressed	Downregulated	Total	Split reads
IDC vs. paired normal	195	180	375	96
IDC vs. apparent normal	38	56	94	25
DCIS vs. apparent normal	29	40	69	24
IDC vs. DCIS	5	7	12	3

We observed antisense RNAs (asRNA) and long intergenic noncoding RNAs (lincRNAs) to be the major lncRNA subtypes differentially expressed among these four groups. asRNAs accounted for 58.9% of total DElncRNAs in IDC compared to paired normal and 55.3% compared to apparently normal samples (Fig. 1E–F). WDR86‐AS1 emerged as a novel antisense lncRNA in our data, whereas ADAMTS9‐AS2 (Li *et al*., 2017; Peng *et al*., 2017) and ST8SIA6‐AS1 (Yang *et al*., 2016a,2016b) have previously been reported in other studies (Fig. 1G–I).

### 3.2 Identification of novel lncRNAs differentially expressed in breast tumors

**Figure 2:**
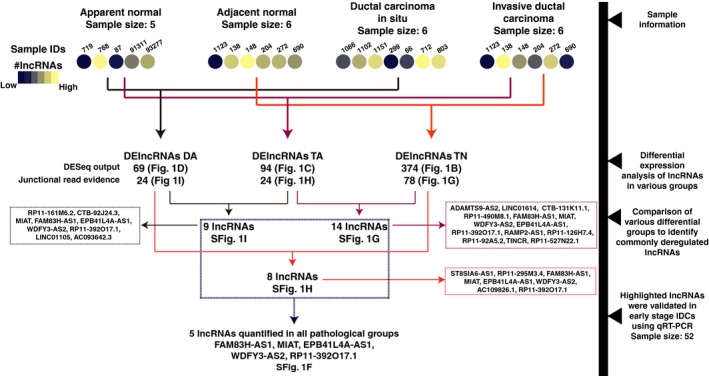
Schematic of lncRNA analysis and cross‐comparison of DElncRNAs in multiple comparison groups.

Dysregulated lncRNAs with evidence of ≥ 2 junction reads in each comparison groups were further investigated (Fig. S1F–I). We identified 21 lncRNAs (11 overexpressed and 10 downregulated) showing a differential expression pattern (Table 2, Fig. 2). Among them, MIAT, FAM83H‐AS1, EPB41L4A‐AS1, WDFY3‐AS2, and RP11‐392O17.1 were commonly deregulated in TN, TA, and DA comparison groups (Fig. 2). Further, LINC01614, RP11‐490M8.1, and CTB‐92J24.3 were novel DElncRNAs identified in early‐stage breast cancer.

**Table 2:** List of DElncRNAs common among various comparison sets

lncRNA	IDC vs. apparent normal	IDC vs. paired normal	DCIS vs. apparent normal	Expression status
MIAT	2.89	1.47	2.72	Overexpressed
FAM83H‐AS1	1.96	1.92	2.01	Overexpressed
LINC01614	5.24	6.1	–	Overexpressed
RP11‐527N22.1	4.2	3.77	–	Overexpressed
TINCR	3.22	4.22	–	Overexpressed
CTB‐131K11.1	2.42	1.96	–	Overexpressed
RP11‐126H7.4	2.22	1.77	–	Overexpressed
LINC01105	3.48	4.04	–	Overexpressed
AC093642.3	2.94	3.39	–	Overexpressed
ST8SIA6‐AS1	–	2.48	3.21	Overexpressed
AC109826.1	–	2.12	2.99	Overexpressed
RAMP‐AS1	−1.38	−1.43	–	Downregulated
ADAMTS9‐AS2	−1.65	−3.31	–	Downregulated
RP11‐490M8.1	−2.32	−1.8	–	Downregulated
RP11‐92A5.2	−3.53	−5.05	–	Downregulated
EPB41L4A‐AS1	−1.55	−1.18	−1.5	Downregulated
WDFY3‐AS2	−1.68	−1.44	−1.65	Downregulated
RP11‐392O17.1	−2.69	−2.72	−2.63	Downregulated
RP11‐161M6.2	−2.44	−2.11	–	Downregulated
CTB‐92J24.3	−2.42	−2.42	–	Downregulated
RP11‐295M3.4	–	−2.79	−2.77	Downregulated

### 3.3 Validation of candidate lncRNA expression in breast tumor and paired normal

**Figure 3:**
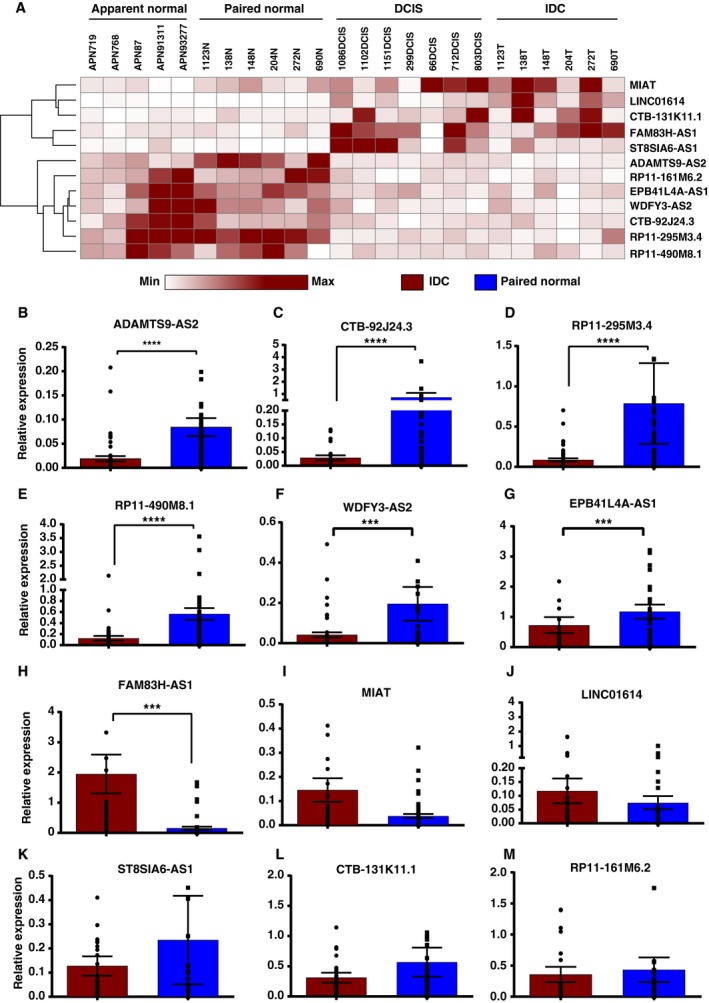
Expression validation of DElncRNAs using qRT‐PCR in cohort of 52 early‐stage breast cancer samples (A) Heatmap of differentially regulated lncRNAs showing expression trend in discovery set of samples. (B) Relative expression of ADAMTS9‐AS2. (C) Relative expression of CTB‐92J24.3. (D) Relative expression of RP11‐295M3.4. (E) Relative expression of RP11‐490M8.1. (F) Relative expression of WDFY3‐AS2. (G) Relative expression of EPB41L4A‐AS1. (H) Relative expression of FAM83H‐AS1. (I) Relative expression of MIAT. (J) Relative expression of LINC01614. (K) Relative expression of ST8SIA6‐AS1. (L) Relative expression of CTB‐131K11.1. (M) Relative expression of RP11‐161M6.2. (B–M are relative expression levels of lncRNA evaluated in validation set of samples); (Wilcoxon sign rank test *P*‐value < 0.0001 = ****, *P* < 0.001 = *** and not indicated for nonsignificant candidates).

We selected 12 candidate lncRNAs (five upregulated lncRNAs: MIAT, FAM83H‐AS1, LINC01614, ST8SIA6‐AS1, and CTB‐131K11.1, and seven downregulated lncRNAs: ADAMTS9‐AS2, EPB41L4A‐AS1, WDFY3‐AS2, RP11‐161M6.2, RP11‐295M3.4, RP11‐490M8.1, and CTB‐92J24.3) for validation using TaqMan™ gene expression assays in *n* = 52 early‐stage IDC samples (Fig. 3A). We observed statistically significant dysregulation of seven out of 12 lncRNAs identified using RNA‐Seq. Among them, ADAMTS9‐AS2 (Fig. 3B) was observed to be the most commonly downregulated lncRNA in tumor tissues (13.59‐fold). We also confirmed significant downregulation of CTB92J24.3 (11.82‐fold), RP11‐295M3.4 (3.5‐fold), RP11‐490M8.1 (3.7‐fold), WDFY3‐AS2 (4.3‐fold), and EPB41L4A‐AS1 (2.09‐fold; Fig. 3C–G). FAM83H‐AS1 was the most significantly overexpressed lncRNA in tumors (8.9‐fold) compared to the paired normal tissues (Fig. 3H). Although, MIAT and LINC01614 were upregulated, they were statistically insignificants (Fig. 3I,J). Whereas, ST8SIA6‐AS1 and CTB‐131K11.1 were found to be down regulated contradicting our RNA sequencing results (Fig. 3K,L). Expression pattern of ST8SIA6‐AS1 and CTB‐131K11.1 in TCGA datasets were similar to the validation results (Fig S3). To evaluate the involvement of receptor status, expression levels of 12 DElncRNAs from validation cohort were correlated with receptors status (ER, PR, HER2; Fig. S4A–D). We observed that MIAT was overexpressed exclusively in samples that were ER+PR+Her2^+^ whereas RP11‐161 M6.2 was overexpressed in ER^−^PR^−^.

### 3.4 ADAMTS9‐AS2 promoter is hypermethylated in breast tumors

**Figure 4:**
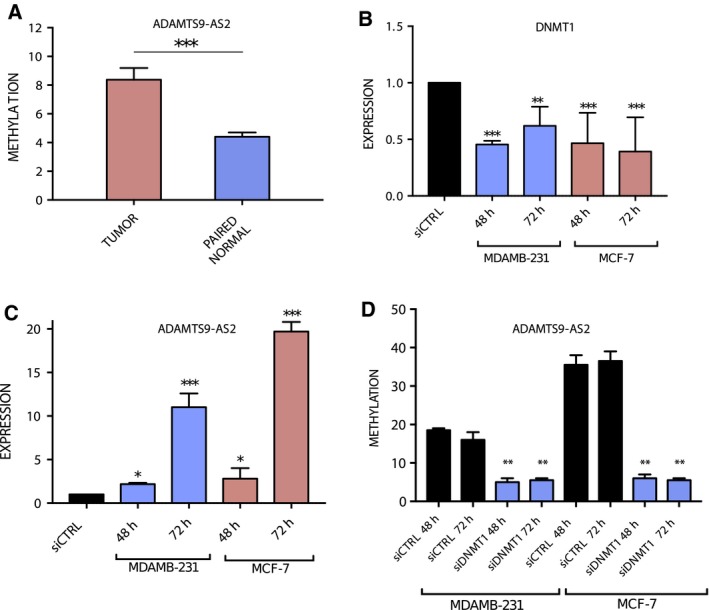
(A) Relative methylation levels of ADAMTS9‐AS2 promoter in tumor vs. paired normal tissue (*N* = 52). (B) Expression levels of DNMT1 with siRNA treatment in MDAMB‐231 and MCF7 cells. (C) Expression of ADAMTS9‐AS2 in MDAMB‐231 and MCF7 cells on DNMT1 knock‐down. (D) Relative methylation levels of ADAMTS9‐AS2 promoter in MDAMB‐231 and MCF7 cells with DNMT1 knock‐down (error bars represent SEM, Student's *t*‐test with two biological independent replicates were used to determine statistical significance; ***P < 0.001, **P < 0.01 and *P < 0.05).

Yao *et al*. (2014) reported the downregulation of ADAMTS9‐AS2 by promoter methylation in gliomas. Hence, methylation levels of the promoter region of ADAMTS9‐AS2 in our validation set of tumor and paired normal samples (*n* = 52) were done using pyrosequencing. We observed a nearly two‐fold (1.9) increase in methylation levels (*P* < 0.0001) in the promoter region (+879 to +929 bp from TSS) of tumor samples compared to paired normal samples (Fig. 4A).

### 3.5 Knock‐down of DNA methyltransferase 1 increases ADAMTS9‐AS2 expression

In order to investigate promoter methylation‐mediated regulation of ADAMTS9‐AS2 expression, DNMT1 was knocked down in MDAMB‐231 and MCF7 using short interfering RNA. The downregulation of DNMT1 led to subsequent overexpression of ADAMTS9‐AS2 by 1.93‐fold (*P* < 0.001) and 2.32‐fold (*P* < 0.001) in MDAMB‐231 and MCF7, respectively (Fig. 4B,C). Loss of promoter methylation was observed using pyrosequencing in DNMT1 siRNA‐transfected MDAMB‐231 (2.6‐fold; *P* = 0.001) and MCF‐7 cells (6.7‐fold; *P* = 0.007; Fig. 4D). These results show that ADAMTS9‐AS2 is overexpressed in both MDAMB‐231 and MCF7 cells following DNMT1 silencing indicating methylation‐mediated suppression of ADAMTS9‐AS2 in breast cancer cells.

### 3.6 Prognostic lncRNAs in early‐stage breast cancer

**Figure 5:**
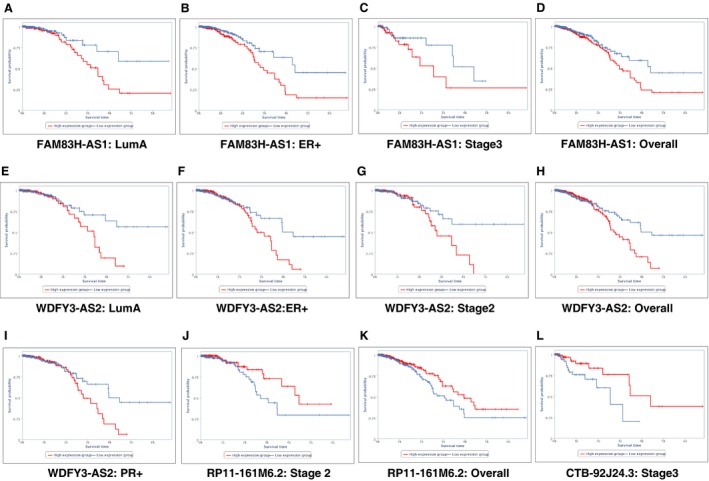
Kaplan–Meier plots derived from TANRIC depicting significant overall poor survival of patients associated with DElncRNAs (A) FAM83H‐AS1 in luminal A molecular subtype. (B) FAM83H‐AS1 in ER+ molecular subtype. (C) FAM83H‐AS1 in stage 3 dataset. (D) FAM83H‐AS1 in overall breast cancer dataset. (E) WDFY3‐AS2 in luminal A molecular subtype. (F) WDFY3‐AS2 in ER+ molecular subtype. (G) WDFY3‐AS2 in stage 2 dataset. (H) WDFY3‐AS2 in overall breast cancer dataset. (I) WDFY3‐AS2 in PR+ molecular subtype. (J) RP11‐161M6.2 in stage 2 dataset. (K) RP11‐161M6.2 in overall breast cancer dataset. (L) CTB‐92J24.3 in stage 3 dataset.

Survival analysis was done to investigate the prognostic potential of candidate lncRNA using TCGA datasets. We observed FAM83H‐AS1 was significantly overexpressed by ~ 4‐fold in TN, TA, as well as DA pairs and its overexpression is associated with overall poor survival in luminal A, ER‐positive tumors, stage 3 datasets, and overall breast tumor datasets irrespective of subtypes (Fig. 5A–D). Overexpression of WDFY3‐AS2 in luminal A, ER‐positive tumors, and breast tumor datasets irrespective of subtypes (Fig. 5E,F,H) is significantly associated with adverse outcomes, whereas downregulation of RP11‐161M6.2 in breast cancer and CTB‐92J24.3 in stage 3 was observed significantly associated with poor overall survival (Fig. 5K). We observed significant association with overexpression of WDFY3‐AS2 (Fig. 5G) and downregulation of RP11‐161M6.2 in stage 2 of breast cancer based on TANRIC analysis indicating them as potential early prognostic markers (Fig. 5G,J).

### 3.7 Co‐expression and pathway analysis

Guilt‐by‐association method was employed to speculate the putative functions of lncRNAs. This approach investigates the association of mRNA expression patterns with lncRNAs using Pearson's correlation analysis. A correlation analysis between DElncRNA‐DEmRNA pairs was done, and only those with PCC ≥ |0.9| were considered significantly co‐expressed. The co‐expressed pairs were filtered for lncRNA with typical junctional read evidence which led to the identification of 2398 pairs consisting of 78 lncRNA and 1097 mRNA between IDC and paired normal samples and 385 pairs consisting of 24 lncRNA and 245 mRNA between IDC and apparent normal samples.

Similarly, 26 pairs were co‐expressed in DCIS vs. apparent normal samples consisting of 11 lncRNA and 26 mRNA and 10 co‐expressed lncRNA‐mRNA pairs in IDC compared to DCIS representing three lncRNA and 10 mRNA (Tables [Link], [Link], [Link], [Link]). Among 2398 co‐expressed lncRNA‐mRNA pairs in IDC vs. paired normal samples, 2225 (92.83%) harbor on different chromosomes (trans‐acting) whereas remaining pairs are cis‐acting. Similarly, 351 (91.64%) out of 383 in IDC vs. apparent normal samples and 23 (85.17%) out of 27 in DCIS vs. apparent normal samples are located on different chromosomes.

Co‐expressed mRNAs were further analyzed using StringDB for network analysis. To augment guilt‐by‐association concept, we further focused on mRNA network that is reported to co‐express irrespective of lncRNA. We observe that partial sets of mRNAs from 22 DElncRNAs in IDC compared to paired normal samples were co‐expressed according to StringDB analysis. After removing disconnected nodes and filtering high confidence nodes from the network, genes co‐expressed with RP11‐142C4.6 (Fig. S5A) were found enriched for extracellular regions (red nodes) and overrepresented for extracellular matrix organization (green nodes) and disassembly (blue nodes) whereas genes co‐expressed with RAMP2‐AS1 were enriched on the cell membrane (red nodes; Fig. S5A,B). Genes co‐expressed with RP11‐701H24.4 were enriched for integral component of membrane (green nodes) and activation of cellular processes (blue nodes; Fig. S5C). In case of PSMB8‐AS1, we observed overrepresentation of immune response and (red nodes) involved in type I interferon signaling pathway (blue nodes; Fig. S5D). We observed enrichment of biological process such as cell division (yellow nodes), cell cycle process (pink nodes), and microtubule cytoskeleton (red nodes) in genes positively co‐expressed with TINCR and negatively co‐expressed with LINC01359 (Figs [Link], [Link]). Interestingly, most genes co‐expressed with PSMB8‐AS1, TINCR, and LINC01359 are also known to co‐express with each other according to StringDB. Using Cytoscape, we were able to segregate the subnetwork of 76 genes potentially governed jointly by TINCR (65 genes) and LINC01359 (55 genes), which resulted in submodules of genes with core histone protein domains (green nodes) and involved in pathways in cancer (blue nodes).

## 4 Discussion

Aberrant expression of lncRNAs is documented in various cancers (Huarte, 2015; Prensner and Chinnaiyan, 2011). In recent years, lncRNAs have gained importance in early detection and better prognosis of tumors (Chandra Gupta and Nandan Tripathi, 2017). Although several lncRNAs associated with breast cancer have been reported previously, studying aberrantly expressed lncRNAs specific to early‐stage breast cancer will provide insight into molecular mechanisms associated with breast cancer development. It will also result in identification of putative markers that might be useful in diagnosis or prognosis of breast cancer. Previous studies have associated altered expression of lncRNAs with specific breast cancer subtypes. For example, HOTAIR is a lncRNA that is highly expressed in HER2+ breast cancers whereas HOTAIRM1 is highly expressed in basal‐like subgroup of breast cancers (Su *et al*., 2014). Luminal A types showed overexpression of LINC00160, and abundance of DSCAM‐AS1 was reported in luminal B subtypes of breast cancer (Jonsson *et al*., 2015; Vu *et al*., 2016). MALAT, lncRNA‐ATB, BC200, XIST, and H19 are some of other lncRNAs frequently associated with breast tumorigenesis and progression (Hansji *et al*., 2014). Functionally important lncRNAs in early‐stage breast cancers are less reported. Our study evaluated the landscape of lncRNA expression in early‐stage breast cancer [IDC (stage I–IIA) and DCIS breast tissues] to identify aberrantly expressed lncRNAs.

The DESeq analysis resulted in identification of 375 DElncRNAs in IDC compared to paired normal samples and 94 DElncRNAs in IDC compared to apparent normal samples. The analysis also identified 69 DElncRNAs in DCIS compared to apparent normal samples. We identified several antisense lncRNAs including ADAMTS9‐AS2, EPB41L4A‐AS1, WDFY3‐AS2, FAM83H‐AS1, ST8SIA6‐AS1, CTB‐92J24.3, and CTB‐131K11.1 that were aberrantly expressed. Twelve candidate lncRNAs that showed significant differential expression were further validated in 52 paired tumor and normal breast samples. We observed significant downregulation of ADAMTS9‐AS2, WDFY3‐AS2, RP11‐295M3.4, RP11‐490M8.1, and CTB‐92J24.3 and significant overexpression of FAM83H‐AS1 in breast cancer. We found ADAMTS9‐AS2 to be significantly downregulated in tumor compared to paired normal breast tissues. ADAMTS9‐AS2 is an antisense transcript originating from the opposite stand coding for ADAMTS9 which is a known inhibitor of angiogenesis and is implicated to have a tumor‐suppressive role. Functional importance of ADAMTS9 in nasopharyngeal and esophageal cancers has been described (Lo *et al*., 2010). ADAMTS9‐AS2 like ADAMTS9 is downregulated in glioblastoma (Yao *et al*., 2014), colorectal cancer (Li *et al*., 2016), bladder cancer, lung adenocarcinoma, and ER+ breast cancers (Li *et al*., 2017). Yao *et al*. have shown that promoter methylation regulates ADAMTS9‐AS2 expression by knocking down DNMT1 in glioma cells. We found that methylation of ADAMTS9‐AS2 controls its expression through correlative DNMT1 knock‐down in MDAMB‐231 and MCF7 cells. Similar results were observed when methylation levels at ADAMTS9‐AS2 promoter were compared between tumors and paired normal tissues using pyrosequencing. We observed DNA methylation‐mediated loss of ADAMTS9‐AS expression in stage I breast cancer. Among other downregulated lncRNAs, WDFY3‐AS2 has recently been reported with TGF‐B‐induced EMT of breast cancer cells through hnRNP‐R modulated positive regulation of STAT3 and WDFY3 (Richards *et al*., 2016). Downregulation of WDFY‐AS2 was found in diffuse glioma and strongly associated with poor prognosis (Wu *et al*., 2018). EPB41L4A‐AS1 (also known as TIGA1) has been shown to be transcribed during growth arrest but has not been extensively studied in cancer to elucidate its role (Yabuta *et al*., 2006). RP11‐161M6.2 was found to be overexpressed in ER/PR−negative and HER2− positive breast cancers in our samples. The finding indicates an association of RP11‐161M6.2 and estrogen receptor and is possibly downregulated in estrogen‐mediated signaling. Similarly, MIAT was dominantly expressed in ER/PR/HER2+ breast cancers samples.

FAM83H‐AS1 was consistently overexpressed in breast tumor samples and overall survival, analysis of TCGA datasets showed poor prognosis of the upregulated group which are in agreement with other studies in breast, colorectal, and lung cancers (Lu *et al*., 2018; Yang *et al*., 2016a,2016c; Zhang *et al*., 2017). Functional studies have demonstrated proliferative potential of FAM83H‐AS1 through MET/EGFR signaling in lung adenocarcinoma and NOTCH1 signaling pathway in colorectal cancer. Overexpression of FAM83H‐AS1 in luminal‐type breast cancer was associated with good prognosis in patients (Yang *et al*., 2016a). Detection of FAM83H‐AS1 expression levels in plasma could be a potential diagnostic and prognostic biomarker for breast cancer.

## 5 Conclusion

In summary, this study has shed light on novel lncRNA and substantiated several previous findings on lncRNA involved in early‐stage breast cancers. We report 375 and 94 lncRNA differentially expressed in tumor samples compared to paired and apparent normal samples, respectively, and 69 DElncRNAs in DCIS compared to apparent normal samples. Seven downregulated and five upregulated lncRNA were further validated to discover significant lncRNA candidate with potential role in breast carcinogenesis. ADAMTS9‐AS2 was one of the lncRNAs consistently downregulated in patient samples, and experimental evidence proved promoter methylation as major cause of ADAMTS9‐AS2 downregulation in breast cancer. Moreover, LINC01614, RP11‐490M8.1, and CTB‐92J24.3 are novel lncRNA reported in our study that has not been associated with breast cancer earlier. Our study also contributes to the existing evidence on MIAT and FAM83H‐AS1 as crucial lncRNA expressed at preliminary stages of breast cancer.

## Conflict of interest

The authors declare no conflict of interest.

## Author contributions

AKDMR, KP, HG, and SM planned the experiments. AKDMR, KP, SKJ, and BM carried out the experiments. VS and SS contributed to surgical excision of tissue samples and histopathological confirmation. AKDMR, KP, AC, HG, and SM contributed to the interpretation of the results. KP and HG contributed to visualizations. AKDMR and SM validated the results. AKDMR and KP wrote the original manuscript. AC, HG, and SM reviewed, edited, and finalized the manuscript. AC, HG, AP, TR, and SM designed the study. All authors provided critical feedback and helped shape the research, analysis, and manuscript.

## Supporting information


**Fig. S1.** Expression pattern of lncRNAs and protein coding genes in various pathological subtype and comparison of DElncRNAs in different groups. (A) Comparative histogram represents relatively lower expression of lncRNAs (blue bars) compared to protein coding genes (grey bars) based on raw read count profile of apparent normal samples (*n* = 5) (B) Comparative histogram represents relatively lower expression of lncRNAs (green bars) compared to protein coding genes (grey bars) based on raw read count profile of paired normal samples (*n* = 6) (C) Comparative histogram represents relatively lower expression of lncRNAs (yellow bars) compared to protein coding genes (grey bars) based on raw read count profile of DCIS samples (*n* = 7) (D) Comparative histogram represents relatively lower expression of lncRNAs (red bars) compared to protein coding genes (grey bars) based on raw read count profile of IDC samples (*n* = 6) (E) PCA using normalized read counts of protein coding genes. Color legend. Apparent normal samples: Yellow, DCIS samples: Purple, Paired normal samples: Green, IDC samples: Red (F) Venn diagram depicting comparison of differential expression analysis group IDC vs. paired normal, IDC vs. apparent normal and DCIS vs. apparent normal samples (G) Venn diagram depicting comparison of differential expression analysis group IDC vs. paired normal and IDC vs. apparent normal samples (H) Venn diagram depicting comparison of differential expression analysis group DCIS vs. apparent normal and IDC vs. paired normal samples (I) Venn diagram depicting comparison of differential expression analysis group IDC vs. apparent normal and DCIS vs. apparent normal samples.

**Fig. S2.** Summary of lncRNA expression profile in IDC vs. DCIS. (A) Volcano plot representing expression pattern in IDC vs. DCIS (B) Heatmap depicting expression trend of differentially expressed gene in IDC vs. DCIS.

**Fig. S3.** LncRNA expression profile in various molecular subtype of breast cancer obtained from TCGA dataset using TANRIC platform (A) RP11‐161M6.2 (B) ADAMTS9‐AS2 (C) CTB‐92J24.3 (D) CTB‐131K11.1 (E) EPB41L4A‐AS1 (F) FAM83H‐AS1 (G) LINC01614 (H) MIAT (I) RP11‐295M3.4 (J) RP11‐490M8.1 (K) ST8SIA6‐AS1 (L) WDFY3‐AS2.

**Fig. S4.** Expression levels of deregulated lncRNAs in various combination of receptors (ER, PR, HER2) positivity in TCGA dataset (A) ER+ or PR+ or Her2+ (B) ER+ or PR+ along with Her2− (C) ER+ and PR− and Her2+ (D) ER− or PR− along with Her2+ (E) Molecular subtype stratification of validation cohort; Red background: Upregulated lncRNAs and Blue background: Downregulated lncRNAs.

**Fig. S5.** High confidence interaction network (score: 0.7) representing differentially expressed mRNA that are known to co‐express with each other as per String analysis and with lncRNA with Pearson correlation coefficient ≥ 0.9 (A) RP11‐142C4.6 (B) RAMP2‐AS2 (C) RP11‐701H24.4 (D) PSMB8‐AS1.

**Fig. S6.** High confidence interaction network (score: 0.7) representing differentially expressed mRNA that are known to co‐express with each other as per String analysis and with lncRNA with Pearson correlation coefficient ≥ 0.9 with TINCR.

**Fig. S7.** High confidence interaction network (score: 0.7) representing differentially expressed mRNA that are known to co‐express with each other as per String analysis and with lncRNA with Pearson correlation coefficient ≤ −0.9 with LINC01359.

**Table S1.** List of clinicopathological features of patients’ tissue samples used in discovery and validation cohort in the study.

**Table S2.** Read alignment statistics and number of genes identified in different samples.

**Table S3.** Complete list of DElncRNAs identified to be differentially expressed in IDC (T) vs. paired normal (N) samples with adjusted *P*‐values < 0.1 in this study along with normalized read counts from individual samples.

**Table S4.** Complete list of DElncRNAs identified to be differentially expressed in IDC (T) vs. apparent normal (APN) with adjusted *P*‐values < 0.1 in this study along with normalized read counts from individual samples.

**Table S5.** Complete list of DElncRNAs identified to be differentially expressed in DCIS vs. apparent normal (APN) with adjusted *P*‐values < 0.1 in this study along with normalized read counts from individual samples.

**Table S6.** Complete list of DElncRNAs identified to be differentially expressed in IDC (T) vs. DCIS with adjusted *P*‐values < 0.1 in this study along with normalized read counts from individual samples.

**Table S7.** Complete list of dysregulated mRNA co‐expressed with dysregulated lncRNAs supported by split reads in IDC vs. paired normal with Pearson correlation coefficient (PCC) ≥ 0.9.

**Table S8.** Complete list of dysregulated mRNA co‐expressed with dysregulated lncRNAs supported by split reads in IDC vs. apparent normal with Pearson correlation coefficient (PCC) ≥ 0.9.

**Table S9.** Complete list of dysregulated mRNA co‐expressed with dysregulated lncRNAs supported by split reads in DCIS vs. apparent normal with Pearson correlation coefficient (PCC) ≥ 0.9.

**Table S10.** Complete list of dysregulated mRNA co‐expressed with dysregulated lncRNAs supported by split reads in IDC vs. DCIS with Pearson correlation coefficient (PCC) ≥ 0.9.

**Table S11.** List of gene expression assays

## Data Availability

Raw sequencing data are available in Sequence Read Archive hosted by National Center for Biotechnology Information (NCBI) search database with accession number PRJNA484546.
